# Public knowledge and awareness of tick-borne pathogens and diseases: A cross-sectional study in Ghana

**DOI:** 10.1016/j.crpvbd.2024.100228

**Published:** 2024-11-04

**Authors:** Theophilus Yaw Alale, Jani J. Sormunen, Joseph Nzeh, Richard Osei Agjei, Eero J. Vesterinen, Tero Klemola

**Affiliations:** a Department of Biology, University of Turku, FI-20014, Turku, Finland; b Biodiversity Unit, University of Turku, FI-20014, Turku, Finland; c Department of Food Science and Technology, Kasetsart University, Bangkok, Thailand; d Department of Health Administration and Education, University of Education, P.O.Box 25, South Campus, Winneba, Ghana

**Keywords:** Awareness, Livestock, Public health, Survey, Tick-borne diseases, Tick-borne pathogens

## Abstract

Tick-borne diseases (TBDs) pose a significant and increasing health threat globally. About 45 tick species have been described from Ghana, located in sub-Saharan Africa, but it is unknown how well-informed local citizens are regarding the risks posed by ticks and TBDs. Utilizing a cross-sectional design with questionnaires, this study assessed the public knowledge and awareness of ticks, tick-borne pathogens (TBPs) and TBDs in Ghana. With demographic background data, we received knowledge responses from 537 respondents across all 16 regions of Ghana through an electronic survey and structured interview questionnaire. Descriptive table statistics were used to tabulate frequencies and percentages of all categorical responses and more closely tested for associations between certain variable pairs. Our results showed that 88% of respondents kept at least one animal species irrespective of being a farmer or not. Of all respondents, over 70% (*n* = 352) were not aware of TBDs in humans while over 50% (*n* = 289) indicated their awareness of TBDs in animals. Interestingly, we found a significant association between age group and awareness of TBDs in animals. Furthermore, the results highlighted the association between domestic animal ownership and increased human-tick encounters. These findings suggest a need for targeted public education on TBDs in Ghana. As Ghana imports livestock, the risk of TBD spread demands attention. Overall, the survey contributes essential insights for veterinary and public health interventions, stressing the urgency of raising awareness and understanding among the public regarding the risks associated with ticks and TBDs.

## Highlights

• Public knowledge of diverse TBDs remains limited, underscoring the need for public education among respondents.
• Online resources form the primary source of TBDs information, especially for younger, educated individuals.
• Older male farmers demonstrated higher awareness of TBDs due to their frequent exposure to livestock management.
• Limited access to veterinary services in rural areas contributes to low awareness of TBDs among respondents.

## Graphical abstract

**Figure:**
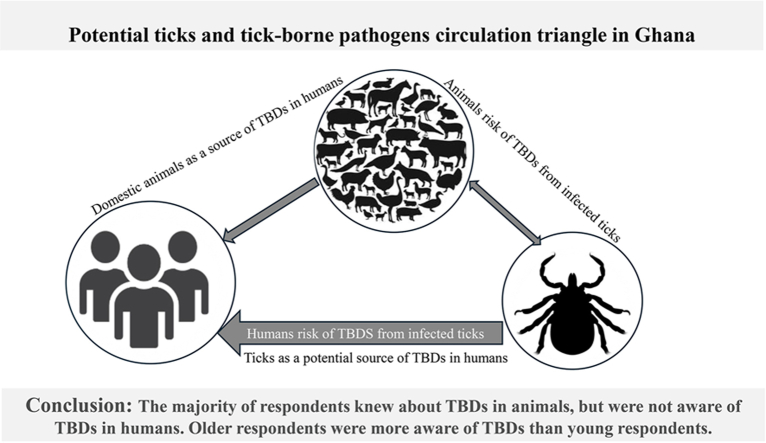

## 1 Introduction

Ticks belong to the order Acari, comprising over 700 species described for Ixodidae and over 200 species identified for Argasidae alone (Guglielmone et al., 2010; Brites-Neto et al., 2015). They are essential ectoparasite arthropods that play a significant role in the transmission of a wide array of tick-borne pathogens (TBPs), impacting both human and animal health globally (Brites-Neto et al., 2015). As vectors, ticks are known for the transmission of various diseases caused by bacteria (e.g. *Coxiella burnetii*), viruses (e.g. Crimean-Congo haemorrhagic fever virus), and protozoans (e.g. *Babesia* spp.), contributing significantly to the global burden of infectious diseases (Araya-Anchetta et al., 2015; Kumar et al., 2019). Globally, the distribution and prevalence of ticks and tick-borne diseases (TBDs) have been influenced by diverse factors, such as climate change, wildlife migration patterns, and human activities such as habitat fragmentation (Gilbert, 2021). These factors may contribute to the dynamic nature of tick populations and their habitats, facilitating the spread of pathogens across different regions (Brites-Neto et al., 2015). For instance, the brown dog tick *Rhipicephalus sanguineus* is widespread and associated with several pathogens affecting dogs and humans worldwide (Gondard et al., 2017; Nimo-Paintsil et al., 2022).

Ticks and TBDs pose substantial challenges to public health and livestock productivity in many parts of Africa (Kivaria, 2006; Akuffo et al., 2016). In eastern and southern Africa, the genera *Amblyomma* and *Rhipicephalus* are prevalent (Horak et al., 2015), while in West Africa, ticks of the genera *Hyalomma* and *Dermacentor* are more commonly found (Akuffo et al., 2016; Nimo-Paintsil et al., 2022; Yessinou et al., 2022; Amoah et al., 2024). The economic impact of TBDs on the agricultural sector is particularly severe, with diseases such as East Coast fever, anaplasmosis, and babesiosis causing significant livestock morbidity and mortality across the continent (Kivaria, 2006; Gondard et al., 2017; Makwarela et al., 2023; Mucheka et al., 2023). For instance, in Tanzania alone, cattle mortality and losses were estimated to be 1.3 million dollars due to theileriosis, anaplasmosis and babesiosis (Kivaria, 2006), while studies involving 912 and 371 tick pooled samples found *Rickettsia* spp. and *Coxiella burnetii* to be the most prevalent tick-borne pathogens in Ghana (Nimo-Painstil et al., 2022; Amoah et al., 2024). Undoubtedly, TBDs remain a significant health threat to most parts of sub-Saharan Africa, including Kenya, Nigeria, Senegal, Ivory Coast, Burkina Faso, and Ghana (Bishop et al., 2023). This regional variation necessitates localized approaches to tick management and disease prevention.

In Ghana, the impact of ticks and TBDs is increasingly being recognized as a significant public health and agricultural concern (Akuffo et al., 2016; Nimo-Paintsil et al., 2022; Amoah et al., 2024). At least 45 tick species have been reported locally (Ntiamoa-Baidu et al., 2004). These species comprise three genera with four species of the family Argasidae, and six genera with 41 species of the family Ixodidae (Ntiamoa-Baidu et al., 2004). The tropical climate, characterized by distinct wet and dry seasons, supports a variety of tick species, including *Rhipicephalus appendiculatus* and *Amblyomma variegatum* (Alkishe et al., 2017; Gilbert, 2021; Lee and Chung, 2023). These ticks are vectors of several pathogens that affect both livestock and humans, such as *Ehrlichia ruminantium*, the causative agent of heartwater disease, and various *Rickettsia* spp. (Mucheka et al., 2023). Domestic animals (cattle, goat, sheep) and pet animals including mainly dogs and cats are the key source of human-biting ticks in Ghana (Brites-Neto et al., 2015; Nimo-Paintsil et al., 2022). As Ghana imports livestock (cattle, sheep, and goats) from neighboring Burkina Faso to supplement local production, the spread of TBDs can be expected to occur (Nimo-Paintsil et al., 2022), and this may likely put local livestock production at risk (Kivaria, 2006). In the veterinary perspective, animals can contract infections through tick bites, while human transmission may also occur through the consumption of unprocessed infected animal products such as milk, blood, meat, and hide (Nimo-Paintsil et al., 2022).

Considering the risks associated with prevalent TBDs in Ghana, the public should be informed about the dangers associated with living in close contact with hosts of ticks, either as pets and companion animals or livestock/farm animals. Studies assessing the knowledge, attitude and practices related to ticks and TBDs have been conducted in some countries in Africa including South Africa (Ngoshe et al., 2023), Kenya (Mutavi et al., 2021), Ethiopia (Tesfaye and Abate, 2023), and Nigeria (Nnabuife et al., 2023). In Ghana, studies assessing the prevalence of ticks and TBDs in livestock exist (Akuffo et al., 2016; Nimo-Paintsil et al., 2022; Amoah et al., 2024), but research specifically focusing on public awareness across different demographic groups is still lacking. This study aimed to assess the public knowledge and awareness of ticks, TBPs and TBDs in Ghana through a questionnaire survey. Considering the limited amount of research focused on public health and veterinary education on ticks and TBDs in Ghana, we hypothesized that public awareness of TBDs in domestic animals and humans would be limited.

## 2 Materials and methods

### 2.1 Study design and target population

**Fig. 1:**
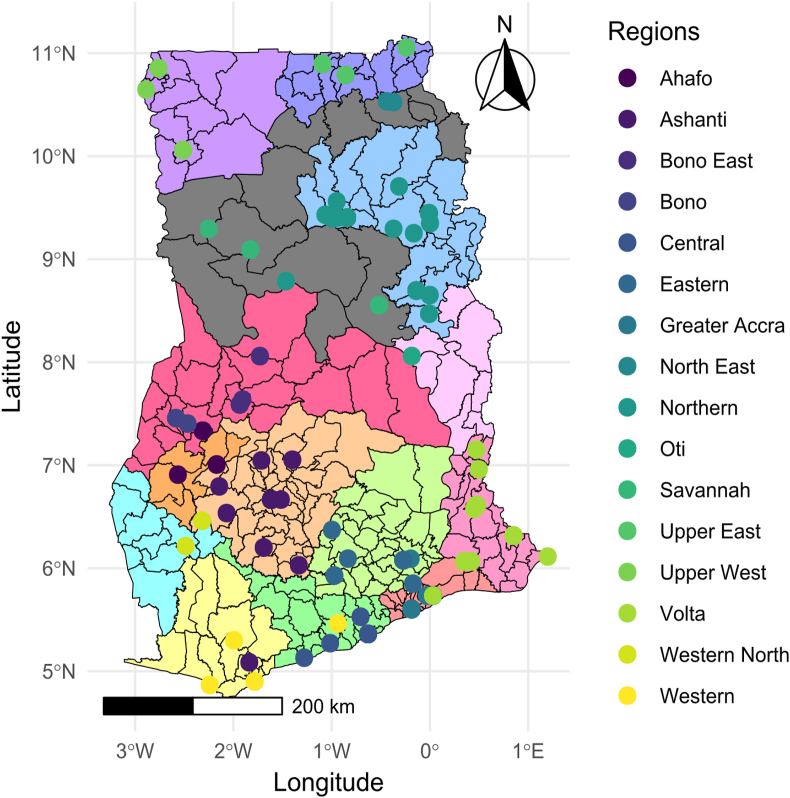
The regional map of Ghana. The color legend shows the geographical regions of Ghana where data were collected from. Circle points indicate locations provided by respondents.

This study employed a cross-sectional survey design using both electronic and printed questionnaires to collect data from a diverse sample population across Ghana. The mixed-method approach allowed for broad coverage and ensured the inclusion of participants with varying access to technology, as well as different regions and demographic groups within Ghana. Respondents were from all 16 regions of Ghana and at least one participant responded from each region (Fig. 1). Eligible respondents were at least 18 years-old. The questionnaires were open from June to September 2022. Overall, 550 respondents from varying backgrounds and professions, covering diverse geographical and demographic groups participated in the study. The sufficient sample size was determined based on a power analysis (using the G*Power software) (Faul et al., 2007; Serdar et al., 2021), by considering the desired significance level (5%), the expected effect size that would generate sufficient statistical power, and the type of statistical test.

We used Google Forms to design the questionnaire to enhance real-time data monitoring and distribution. The questionnaire was designed to capture information on: (i) demographic details (age, gender, education background, occupation, region); (ii) knowledge of ticks and tick-borne diseases; (iii) sources of information about ticks and tick-borne diseases; (iv) attitudes towards ticks and tick prevention practices; and (v) practices related to tick control and prevention. All questions were written in English. However, in areas where respondents could not understand English, interviews were conducted in local languages during administration to ensure comprehensibility and inclusivity. Both close-ended questions and open-ended questions were included. Overall, 10 questions were directed to respondents’ knowledge and awareness of ticks and TBDs (see Supplementary file S1).

### 2.2 Participantsʼ recruitment and data collection tools

For participants in rural communities, local extension agents were used to facilitate the recruitment of survey respondents, while electronic questionnaire links were distributed *via* social media (WhatsApp and Facebook). To encourage participation, reminders were sent periodically. This method was efficient and allowed smooth data collection from respondents who had internet access. Responses submitted through electronic questionnaires were automatically recorded on Google Cloud, minimizing the risk of data entry errors and facilitating real-time data monitoring. Printed responses were manually entered into a digital database immediately by field facilitators to ensure accuracy and consistency in data entry.

### 2.3 Statistical analysis

The data were analyzed using SAS statistical software (Enterprise Guide ver. 8.3 interface). We used descriptive table statistics to tabulate frequencies and percentages of all categorical responses and more closely tested for associations between certain pairs of these categorical variables, always including a demographic variable and a variable related to ticks, TBDs or TBPs. Chi-square test for independence was used to test for associations between demographic factors and awareness levels (chi-square analyses of contingency tables).

## 3 Results

### 3.1 Respondents’ demographic characteristics

After four months (June–September 2022) of questionnaire administration and data gathering, 550 responses were received of which 537 (533 with all demographic fields completed) were retained after filtering out responses with missing data. Respondents’ ages ranged from 18 to over 55 years. A large proportion of respondents (49%) were university students, while 22% were farmers. Demographic characteristics including gender, age group, level of education, and professional qualification of respondents are summarized in Table 1.

**Table 1:** Demographic characteristics of participating respondents in the study.

Variable	Category	Frequency (%)
Age group (years)	18–24	222 (41.7)
	25–34	184 (34.5)
	35–44	65 (12.2)
	45–54	45 (8.4)
	≥ 55	17 (3.2)
Gender	Male	333 (62.5)
	Female	200 (37.5)
Level of education	Basic education	78 (14.6)
	High school education	38 (7.1)
	University education	362 (67.7)
	No formal education	57 (10.7)
Profession	Farmer	118 (22.1)
	Trader	27 (5.1)
	Health worker	29 (5.4)
	Teacher	43 (8.0)
	Student	264 (49.4)
	Other	54 (10.1)

### 3.2 Respondents’ awareness of TBDs

We used three questions (Table 2) to evaluate respondent’s awareness of ticks and TBPs/TBDs. For questions concerning domestic animals as TBD carriers, the majority (58.4%, *n* = 289) of respondents (*n* = 495) answered “yes”, citing that animals often got sick after ticks were found attached to them, while 18.4% (*n* = 91) and 23.2% (*n* = 115) said “no” they have not heard of it or “do not know” that ticks can transmit TBDs to the animals (Table 2). Of the same number of respondents, a significant proportion (71.1%, *n* = 352) had not heard of TBDs in humans. Only 21.6% (*n* = 107) were aware of TBDs in humans (Table 2).

**Table 2:** Respondents’ awareness of potential health threats posed by ticks to humans and animals.

Question	Response	Frequency	Percentage
Do you think ticks can transmit disease pathogens to animals?	Yes	289	58.4
	No	91	18.4
	I do not know	115	23.2
Have you heard of tick-borne pathogen/disease in humans before?	Yes	107	21.6
	No	352	71.1
	I do not remember	36	7.3
Do you think humans can get diseases from tick bites?	Yes	236	47.6
	No	260	52.4

**Fig. 2:**
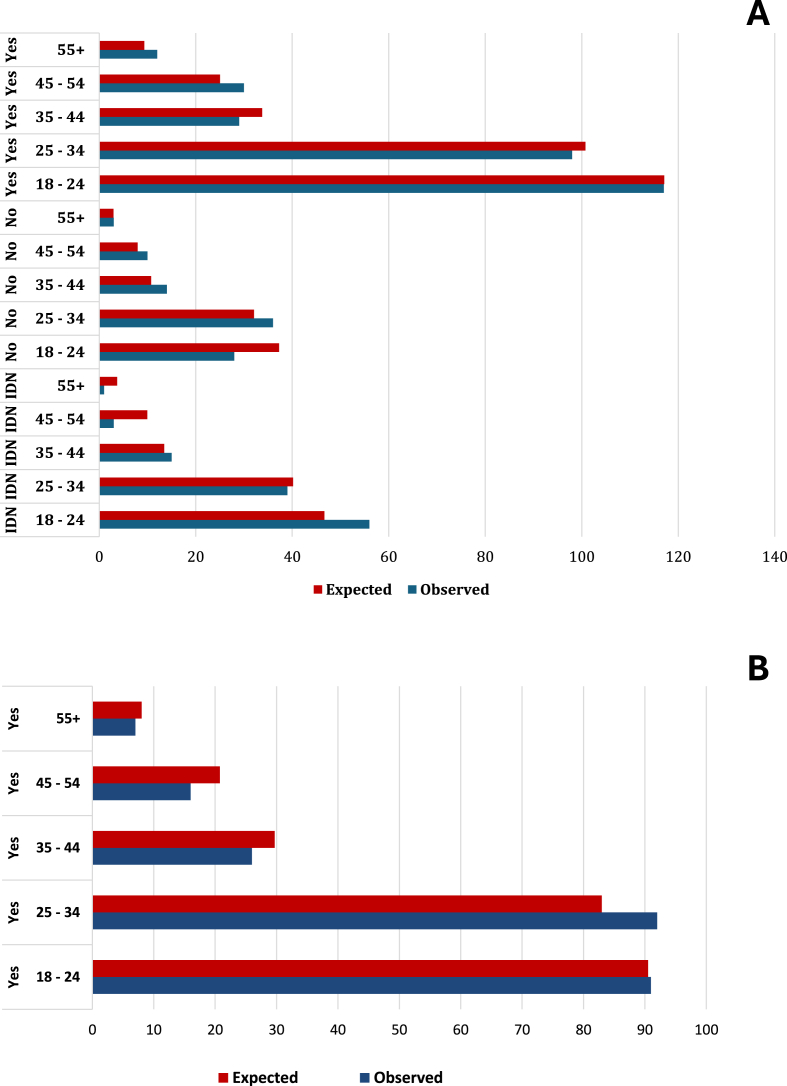
**A** Associations between respondentsʼ age and awareness of TBDs in animals. Respondents of 45–54 and 55+ years age classes were more aware (answer “Yes” with observed frequency > expected frequency) that domestic animals can get diseases from tick bites compared to younger age classes. *Abbreviation*: IDN, “I do not know”. **B**Association between respondents’ age and TBDs awareness of human diseases. There was no significant association between age and TBDs awareness. Observed frequencies of “Yes”/“No” answers did not differ sufficiently from expected frequencies.

**Fig. 3:**
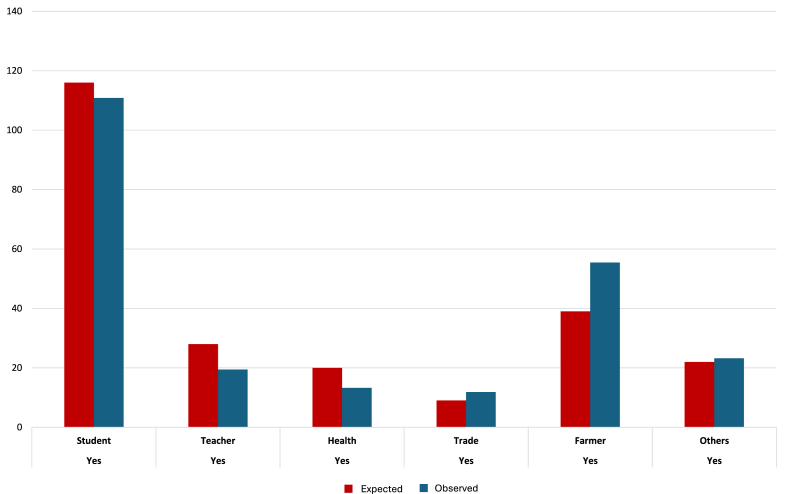
Association between the professional qualification of respondents and the question “Have you heard of tick-borne pathogens and diseases?” (answer “Yes” or “No”). Farmers were more aware of TBPs and TBDs compared to other professions (*χ*^2^ = 59.14, *df* = 5, *P* < 0.0001).

We found a statistically significant association (*χ*^2^ = 15.7, *df* = 8, *P* = 0.05) between the respondent’s age group and his/her awareness of domestic animals as carriers of TBDs (Fig. 2A). However, no such association between age group and awareness of TBDs in humans was observed (*χ*^2^ = 5.0, *df* = 4, *P* = 0.28; Fig. 2B). Moreover, we also found a significant association (*χ*^2^ = 59.14, *df* = 5, *P* < 0.0001) between professional qualification and TBDs awareness of both human and animal diseases (Fig. 3). In addition, we found significant associations between respondents’ level of education (*χ*^2^ = 24.86, *df* = 6, *P* = 0.0004), gender (*χ*^2^ = 9.36, *df* = 2, *P* = 0.009) and their awareness of TBDs in both humans and animals respectively (Supplementary file S2: Figs. S3 and S9). Generally, males were more aware of TBPs compared to females, while those with a university education were more aware of TBDs/TBPs (*χ*^2^ = 53.90, *df* = 3, *P* < 0.0001) (Supplementary file S2: Fig. S4).

### 3.3 Domestic animals as sources of TBDs

To assess the possible sources of TBDs, we collected information about the domestic animals kept by respondents as these could be potential sources of TBDs. Only 11.6% did not keep any animal, while the remaining 88.4% kept at least one domestic animal as well as snails and rabbits (Supplementary file S2: Table S4). The most frequent animals reported were chickens (15.1%), dogs (14.2%), and cats (9.1%). For all animals reported, 7.8% of respondents kept both cats and dogs while 7.5% also kept cats and chickens. Among large ruminants kept by respondents were cattle (0.6%), sheep, and goats (0.4%).

We evaluated respondents’ source information about TBPs/TBDs. Only 298 participants provided answers to the question “from where you heard about TBDs”. Overall, 22.2%, being the highest, chose veterinary personnel, followed by 20.1% citing internet sources, and then 16.8% and 16.4% referring to mass media (TV and radio) and friends or family members, respectively. Interestingly, less than 1% of all respondents cited newspapers or any print media as information sources (Supplementary file S2: Table S5).

Many of respondents (46.0%) reported that they have never received veterinary services, while 26.0%, 19.0%, and 9.0% received veterinary services occasionally, sometimes, and often, respectively (Supplementary file S2: Table S6). Less than 50% of respondents (*n* = 221) answered the question related to the type of TBDs they were aware of. Reported TBDs included bacterial [Lyme disease (*n* = 47) and rickettsiosis (*n* = 5)] and viral [TBEv (*n* = 13) and CCHFv (*n* = 25)] diseases as well as bovine protozoans (*n* = 17) (Supplementary file S2: Table S7). Interestingly, 37.0% of the respondents (n = 82) did not know about any of the TBDs presented in the questionnaire nor indicated any other.

## 4 Discussion

The results of our study shed light on the levels of awareness among respondents regarding ticks, tick-borne diseases (TBDs), and tick-borne pathogens (TBPs) in Ghana. Notably, almost half of the respondents (49%) identified as university students, indicating a strong presence of the younger demographic group, often associated with academic settings. Additionally, the substantial representation of farmers highlights the inclusion of individuals from the agricultural sector. This diversity of inclusion is crucial for achieving comprehensive insights, considering potential variations in experiences and perspectives based on age, education, gender, and professional qualifications (Balkhy et al., 2010; Almutairi et al., 2015).

In exploring demographic associations, the study identified a significant link between age and awareness of domestic animals as TBP reservoirs (Nimo-Painstil et al., 2022). We observed that respondents who were at least 35 years-old and were farmers knew a lot about ticks and TBDs. This finding indicates the close interaction between the type of animal they keep and the surroundings where ticks may likely be found. Again, we noticed from their verbal commentaries that their long years of experience with the animals enabled them to identify what could be the source of the disease despite the lack of veterinary care (Ngoshe et al., 2023). This observation highlights the need for targeted education campaigns across different age groups. Moreover, gender, level of education, and professional qualifications were found to have strong associations with awareness of TBPs/TBDs in both humans and domestic animals (Gondard et al., 2017). Respondent farmers seemed to be more aware of tick-borne diseases than other professionals. This could be due to their frequent exposure to ticks through outdoor work and livestock rearing, which increases their risk of infection. Most of them often have direct contact with their animals, making them more knowledgeable about certain animal diseases (Chakraborty et al., 2023). Additionally, agricultural health campaigns and veterinary extension services frequently target farmers, reinforcing their awareness. For most of these people, their economic powers depend on healthy livestock keeping them informed about these diseases, unlike other professionals who face lower risks and may be unconcerned. Our findings underscore existing gaps in public awareness of TBDs, emphasizing the necessity for educational initiatives tailored to specific demographic groups. Enhancing knowledge about the transmission dynamics of TBPs/TBDs, especially between ticks and domestic or companion animals (Walker, 2017; Saleh et al., 2021), could play a crucial role in disease prevention and control, as rural households, farmers, and slaughterhouse workers may have direct contact with infected livestock (Akuffo et al., 2016; Amoah et al., 2024).

The observed positive association between gender, age, and awareness of tick-borne pathogens in Ghana can be explained by differences in exposure, roles, and access to information. Males are often more aware due to their direct involvement in farming and herding, which increase their risk of encountering ticks. Adult males may have greater awareness due to accumulated life experience, participation in community extension meetings, veterinary campaigns, while younger individuals may have less exposure to such information. These factors combined contribute to the observed association between gender, age, and pathogen awareness. One notable observation was the relatively poor recognition of various types of TBDs of veterinary and public health importance, encompassing bacterial (exemplified by rickettsial diseases), viral (CCHFv), and protozoan diseases (Akuffo et al., 2016; Nimo-Paintsil et al., 2022; Amoah et al., 2024). This lack of recognition further highlights the lack of public education and access to extension/veterinary services for local livestock and peasant farmers among the respondents. A considerable proportion (37%) of respondents expressed a lack of knowledge about any of the TBDs presented in the questionnaire, suggesting potential gaps in public awareness. These findings reveal the importance of targeted education and awareness campaigns to bridge existing gaps and enhance public understanding of the diverse spectrum of TBDs. Efforts to increase awareness should not only focus on well-known TBPs/TBDs but also extend to lesser-known viral, protozoan, and bacterial diseases. This limited understanding and awareness levels provide valuable insights for public health initiatives aimed at improving knowledge and preventive practices related to TBDs.

Understanding the dynamics of information sources is crucial for developing targeted and effective public health campaigns (Huo et al., 2019). The investigation into respondents’ sources of knowledge about TBDs yielded valuable insights into the diverse channels through which individuals acquire information on these pathogens and associated diseases in Ghana. The predominant source identified by respondents was veterinary personnel, emphasizing the significance of professional guidance in shaping public awareness of TBDs. Internet sources closely followed, indicating how participants rely on online platforms for information. This finding complements the dependence of young people (university students) on the Internet as a key source of information (Huo et al., 2019). These findings emphasize the importance of tailored educational strategies that recognize the varied channels through which individuals seek information about TBDs. The dominance of veterinary personnel suggests the critical role that healthcare professionals can play in disseminating accurate information and fostering public awareness (Janke et al., 2021). By recognizing the influential roles of veterinary professionals and online platforms, health educators can strategically focus efforts to enhance public knowledge and awareness of TBDs. Moreover, the substantial reliance on internet sources suggests the need for accessible and reliable online platforms for disseminating information about TBDs. As society increasingly turns to digital platforms for information, public health interventions may benefit from leveraging online resources to reach broader audiences effectively.

Our findings indicate that a high proportion of respondents keep pets/domestic animals and may thus encounter tick-infected animals, and consequently potential TBP carriers. The coexistence of multiple animals in households, as indicated by respondents keeping both cats and dogs or cats and chickens, suggests complex interactions that could influence the transmission dynamics of TBPs (Akuffo et al., 2016; Saleh et al., 2019). Interestingly, a considerable proportion of respondents (46%) reported never having received veterinary services. This finding underscores potential gaps in veterinary care and raises concerns about the management of the health of domestic animals, which may have implications for the spread of TBDs. However, the varying levels of veterinary service utilization, with 26%, 19%, and 9% receiving services occasionally, sometimes, and often, respectively, indicate a spectrum of engagement with veterinary care. Our results suggest that promoting awareness of TBDs should extend beyond human health to encompass responsible animal husbandry practices. Educational initiatives focusing on the role of pets and domestic animals as potential sources of TBPs/TBDs, along with the importance of regular veterinary check-ups, could contribute to a more comprehensive understanding among the public. Livestock farming forms a critical component of Ghanaʼs economy, making close interaction between livestock, wildlife, and humans inseparable. This close association may likely facilitate the transmission of tick-borne pathogens across different host species, compounding the challenge of disease control. Moreover, the lack of comprehensive surveillance systems and limited public awareness further exacerbate the risks associated with tick bites and pathogen transmission.

We acknowledge the limitations of this study including the limited sample size that may affect the generalization of these findings. Therefore, the findings from this study may represent a minor group of participants, hence this study may be carried out with a more representative sample size to be able to generalize the results.

## 5 Conclusions

Our findings highlight the interconnectedness of human and animal association in the context of TBDs. Despite the evident risk posed by prevalent TBPs, awareness among most of the respondents remains limited. Our study also identified a considerable number of survey participants keeping livestock and pets but were without veterinary care and had not heard of TBPs/TBDs in either humans or animals. These results bring to light a concerning gap in public knowledge regarding ticks and TBDs in Ghana. This calls for efforts to enhance awareness and promote responsible animal husbandry practices, as this can contribute to the prevention and control of TBDs in both human and animal welfare. Furthermore, the co-existence of humans with diverse domestic animals coupled with limited access to veterinary services, presents opportunities for targeted public health interventions. To ensure improved awareness creation and knowledge about TBDs and TBPs in Ghana will require *Public Health Education*: Implement targeted public health education campaigns to raise awareness about ticks, tick-borne diseases, and preventive measures; *Proactive Veterinary Services*: Strengthen veterinary services to monitor and control tick infestations among domestic animals and pets; *Surveillance and Research*: Establish a comprehensive tick surveillance system to monitor tick populations, prevalence of tick-borne pathogens, and emerging trends; *International Collaboration*: Foster international collaboration on tick-borne disease research, surveillance, control efforts, and exchange knowledge and best practices with neighboring countries facing similar challenges. The collective efforts of the government, healthcare providers, educational institutions, and communities are vital for creating a well-informed and resilient society in the face of climate change and global health risks.

## CRediT authorship contribution statement

**Theophilus Yaw Alale:** Conceptualization, Methodology, Formal analysis, Visualization, Data curation, Writing – original draft, Writing – review & editing. **Jani J. Sormunen:** Resources, Data curation, Supervision, Project administration, Writing – review & editing, Funding acquisition. **Joseph Nzeh:** Conceptualization, Methodology, Data curation, Formal analysis, Visualization, Writing – review & editing. **Richard Osei Agjei:** Conceptualization, Methodology, Data curation, Formal analysis, Visualization. **Eero J. Vesterinen:** Resources, Data curation, Supervision, Project administration, Funding acquisition, Writing – review & editing. **Tero Klemola:** Methodology, Formal analysis, Investigation, Supervision, Data curation, Writing – review & editing.

## Ethical approval

No animal experimentation was carried out in this study. All data came from questionnaires administered to human participants. Participants were informed about the purpose of the study and how the data will be used. Respondents gave their consent to participate in the study. Personal identifiers were removed from the dataset to ensure anonymity. The unprocessed data has been stored securely and only accessible to the research team. Based on the guidelines for the ethical principles of research with human participants and ethical review in human science in Finland, and the Medical Research Act (488/1999), this study design did not require ethical review and approval.

## Data availability

The data that supporting the conclusions of this article are included within the article and its supplementary files. Raw data are not publicly available due to privacy concerns.

## Funding

This work was supported by the University of Turku Foundation (grant number 081166), the 10.13039/501100003125Finnish Cultural Foundation, and the Sakari Alhopuro Foundation.

## Declaration of competing interests

The authors declare that they have no known competing financial interests or personal relationships that could have appeared to influence the work reported in this paper.

## Data Availability

The data that supporting the conclusions of this article are included within the article and its supplementary files. Raw data are not publicly available due to privacy concerns.

## Appendix A Supplementary data

Supplementary file S1 Questionnaires. Supplementary file S1
