# Supplementary material for: Public knowledge and awareness of tick-borne pathogens and diseases: A cross-sectional study in Ghana
Source: Curr Res Parasitol Vector Borne Dis. 2024 Nov 4;6:100228. doi: 10.1016/j.crpvbd.2024.100228 (PMC11570981; doi:10.1016/j.crpvbd.2024.100228)
Supplement: Supplementary file S1 — Questionnaires. [file mmc1.pdf]

TICKS AND TICK-BORNE PATHOGENS: A SURVEY

We are a group of Researchers (Finland & Ghana) conducting a study on public knowledge, awareness and attitude towards ticks and tick-borne pathogens and diseases in Ghana. Kindly spare 10-15 minutes of your time to respond to these questions. Your anonymity is assured. Thank you

* Indicates required question

1. I agree to participate in this study and understand that all my answers will only be used for scientific purposes. *

   I agree to the processing of my personal data in accordance with the information provided here.
   I am aware that the data will be published in anonymous form to promote transparency in science...

   Mark only one oval.

   - Agree
   - Do not agree

2. Enumerator's name:

   ____

3. Current location: and Town/Community

   ____

4. Region in Ghana

Mark only one oval.

- Ashanti region
- Ahafo region
- Greater Accra region
- Volta region
- Western region
- Western North region
- Oti region
- Central rgion
- Upper East rgion
- North_East region
- Northern region
- Savannah region
- Upper West rgion
- Bono region
- Bono East region
- Eastern region

Section 1.1 Respondent's information

Tick all that are applicable and answer accordingly

5. Your Age

Mark only one oval.

- 18-24
- 25-34
- 35-44
- 45-54
- 55-64
- 65 and Over

6. 2. Sex

Mark only one oval.

- Male
- Female

7. 3. What is your professional qualification

Mark only one oval.

- Farming
- Trading
- Teacher
- Health worker
- Student
- Others, Please, specify:

8. 4. what is your level of education?

Mark only one oval.

- No formal education
- Basic Education/Junior high school
- Techinical/Senior High school
- University degree/Higer education qualification
- Apprenticeship/vocational training
- Adult education

9. 1.2.1 Which of the following animals do you keep at home? Choose all that apply

Check all that apply.

- [ ] Dog(s)
- [ ] Cat(s)
- [ ] Chicken
- [ ] Goat/Sheep
- [ ] Cattle
- [ ] None
- [ ] Other: ____________________

Section 1.2: Tick abundance, populations and health impact awareness.

Assessment of public knowledge on ticks and tick-borne pathogens and diseases

10. 1.2.2 Why do you keep this/these animal(s)? Choose all that apply

Check all that apply.

- [ ] As pets
- [ ] For food/meat
- [ ] For sale
- [ ] Security
- [ ] Religious/cultural reasons
- [ ] Other: ____________________

11. 1.2.3 Have you seen a tick around your house?

Mark only one oval.

- ( ) Yes
- ( ) No: if no, skip the next question

12. 1.2.4 Where did you find the tick?

Mark only one oval.

- On the house floor
- In the bushes/lawns/hedges around
- Attached to animal's skin
- Other: ______________________

13. 1.2.5 If attached to animal's skin: which part of the animal was the tick attached to? Choose all that apply

Check all that apply.

- Inside/outside the ear region
- On the head and neck region
- Between the legs towards reproductive organs
- Backside/ belly region
- Between the hooves
- Other: ______________________

14. 1.2.6 What time of the day do you normally see ticks around? Choose all that apply

Check all that apply.

- Morning
- Afternoon
- Evening
- Everytime

15. 1.2.7 In which season of the year are tick commomly found?

Mark only one oval.

- Rainy season
- Dry season
- Throughout the year
- Not sure

16. 1.2.8 Where do you think the ticks come from?

Mark only one oval.

- From the surrounding bushes
- Free ranging animals bring them from the bush
- Don't know

17. 1.2.9 What do you do when you notice a tick on your animal

Check all that apply.

- [ ] I remove and kill it immediately
- [ ] I remove and throw away without killing it
- [ ] I apply acaricide/insecticide on the animal and allow it to go without removing the tick
- [ ] I do nothing because they are not harmful to the animal

18. 1.2.10 Does any of your animal get sick

Mark only one oval.

- Yes
- No
- Maybe

19. 1.2.11 Have you heard of tick-borne pathogens/diseases?

Mark only one oval.

- Yes
- No
- Maybe

20. 1.2.12 From where did you hear about tick-borne pathogens and diseases? Choose all that apply

Check all that apply.

- [ ] From the media(Tv and radio)
- [ ] I read fro the news paper
- [ ] From the internet
- [ ] From a veterinary expert
- [ ] From a friend/family member

21. 1.2.13 Do you think that the tick causes diseases to the animal?

Mark only one oval.

- Yes, because the animals alway get sick whenever ticks are attached to it
- No, because the animal has never been sick even when ticks are found attched to it
- I don't know

22. 1.2.14 How often do you get veterinary services for your animals?

Mark only one oval.

- Never
- Occasionally (when I suspect something is wrong)
- Sometimes (only when they are sick)
- Often (regular routine checks)

23. 1.2.15 What type of diseases/infections/symptoms are the animals often diagnosed of? Choose all that apply

Check all that apply.

- [ ] Diarrhoea/watery droppings
- [ ] Running nose and teary eyes
- [ ] Dullness and inability to eat/Isolation from other animal and people
- [ ] Rushes and sores on the skin
- [ ] Pregnancy abortion
- [ ] None othe above
- [ ] Other: ____________

24. 1.2.16 What type of tests are conducted to diagnose the disease on the animal?

Mark only one oval.

- ( ) Blood test
- ( ) Physical examination and prescribe drug for the animal
- ( ) No test is done. Veterinary officer just tells us the drug to buy for treatment
- ( ) No test is done. I just go to the pharmacy and buy a drug for my sick animals

25. 1.2.17 What kind of treatments are given to the animals when they are sick? Choose all that apply

Check all that apply.

- [ ] Antibiotics
- [ ] Antiviral vaccination
- [ ] Something to stop the diarrhoea
- [ ] Pain killers
- [ ] I only apply ointments to the wound and rushes
- [ ] I don't know about the drug

2.0 Tick, tick-borne diseases and humans

Public health wareness on tick-borne pathogens and diseases

26. 2.1 Have you been bitten by a tick before?

Mark only one oval.

- Yes. proceed to the next question
- No. skip the next 2 questions!
- Maybe

27. 2.2 What were the symptoms/signs of the tick bite? Choose all that apply

Check all that apply.

- [ ] Itching and irritations
- [ ] Swelling and rashes around the bitten area
- [ ] Fever and headache
- [ ] No symptoms at all

28. 2.3 What action did you take after taking the tick bite?

Mark only one oval.

- I went to the doctor/pharmacy to get tested for possible infections
- I did nothing about it because it did not hurt
- I hate tick but they do not cause any harm to human
- Other: ______________

29. 2.4 Have you heard of tick-borne pathogens and disease in humans before?

Mark only one oval.

- Yes. proceed to the next question
- No, I have never heard of it. skip the next question
- I don't remember

30. 2.5 What type of tick-borne pathogens/diseases do you know of? Choose all that apply

Check all that apply.

- [ ] Bacteria (e.g. Borelia disease/lyme disease)
- [ ] Viral diseases (e.g tick-borne encephalitis-TBE, Cremean-congo virus-CCHV)
- [ ] Protozoa diseases
- [ ] Rickettsia diseases
- [ ] None of the above
- [ ] Other: ______________

31. 2.6 Do you think that humans can get diseases from tick bite?

Mark only one oval.

- ( ) Yes
- ( ) No
- ( ) Maybe

32. 2.7 Do you think you have ever gotten tick-borne diseases before without knowing?

Mark only one oval.

- ( ) Yes
- ( ) No
- ( ) Maybe

33. 2.8 Have you ever tested for tick-borne infections before?

Mark only one oval.

- ( ) Yes
- ( ) No

34. 2.9 Have you or any of your family members been diagnosed of tick-borne diseases before?

Mark only one oval.

- Yes
- No
- I have never heard of it

35. 2.10 If yes, what were the symptoms you experienced? Choose all that apply

Check all that apply.

- [ ] Diarrhoea/frequent watery stool
- [ ] Fever and headache
- [ ] Rashes and swelling on my body area
- [ ] Dizziness and loss of appettite
- [ ] No symptoms at all
- [ ] Other: ______________________

36. 2.11 Have you ever received treatments for suspected tick-borne diseases/infection)

Mark only one oval.

- Yes
- No

37. 2.12 If yes, what kind of treatment were you given/take?

Mark only one oval.

- Herbal medicine/formula
- Antibiotics prescribed by a doctor
- I did not take any medicine
- Self medication
- Pain killers

38. I agree to participate in this study and understand that all my answers will only be used for scientific purposes. *

I agree to the processing of my personal data in accordance with the information provided here.
I am aware that the data will be published in anonymous form to promote transparency in science...

Mark only one oval.

- Agree
- Do not agree

39. Enumerator's name:

____

40. Current location: and Town/Community

____

41. Region in Ghana

Mark only one oval.

- Ashanti region
- Ahafo region
- Greater Accra region
- Volta region
- Western region
- Western North region
- Oti region
- Central rgion
- Upper East rgion
- North_East region
- Northern region
- Savannah region
- Upper West rgion
- Bono region
- Bono East region
- Eastern region

Section 1.1 Respondent's information

Tick all that are applicable and answer accordingly

42. Your Age

Mark only one oval.

- 18-24
- 25-34
- 35-44
- 45-54
- 55-64
- 65 and Over

43. 2. Sex

Mark only one oval.

- Male
- Female

44. 3. What is your professional qualification

Mark only one oval.

- Farming
- Trading
- Teacher
- Health worker
- Student
- Others, Please, specify:

45. 4. what is your level of education?

Mark only one oval.

- No formal education
- Basic Education/Junior high school
- Techinical/Senior High school
- University degree/Higer education qualification
- Apprenticeship/vocational training
- Adult education

46. 1.2.1 Which of the following animals do you keep at home? Choose all that apply

Check all that apply.

- [ ] Dog(s)
- [ ] Cat(s)
- [ ] Chicken
- [ ] Goat/Sheep
- [ ] Cattle
- [ ] None
- [ ] Other: ______________________

Section 1.2: Tick abundance, populations and health impact awareness.
Assessment of public knowledge on ticks and tick-borne pathogens and diseases

47. 1.2.2 Why do you keep this/these animal(s)? Choose all that apply

Check all that apply.

- [ ] As pets
- [ ] For food/meat
- [ ] For sale
- [ ] Security
- [ ] Religious/cultural reasons
- [ ] Other: ______________________

48. 1.2.3 Have you seen a tick around your house?

Mark only one oval.

- ( ) Yes
- ( ) No: if no, skip the next question

49. 1.2.4 Where did you find the tick?

Mark only one oval.

- On the house floor
- In the bushes/lawns/hedges around
- Attached to animal's skin
- Other: ____________________

50. 1.2.5 If attached to animal's skin: which part of the animal was the tick attached to? Choose all that apply

Check all that apply.

- [ ] Inside/outside the ear region
- [ ] On the head and neck region
- [ ] Between the legs towards reproductive organs
- [ ] Backside/ belly region
- [ ] Between the hooves
- [ ] Other: ____________________

51. 1.2.6 What time of the day do you normally see ticks around? Choose all that apply

Check all that apply.

- [ ] Morning
- [ ] Afternoon
- [ ] Evening
- [ ] Everytime

52. 1.2.7 In which season of the year are tick commomly found?

Mark only one oval.

- Rainy season
- Dry season
- Throughout the year
- Not sure

53. 1.2.8 Where do you think the ticks come from?

Mark only one oval.

- From the surrounding bushes
- Free ranging animals bring them from the bush
- Don't know

54. 1.2.9 What do you do when you notice a tick on your animal

Check all that apply.

- [ ] I remove and kill it immediately
- [ ] I remove and throw away without killing it
- [ ] I apply acaricide/insecticide on the animal and allow it to go without removing the tick
- [ ] I do nothing because they are not harmful to the animal

55. 1.2.10 Does any of your animal get sick

Mark only one oval.

- Yes
- No
- Maybe

56. 1.2.11 Have you heard of tick-borne pathogens/diseases?

Mark only one oval.

- Yes
- No
- Maybe

57. 1.2.12 From where did you hear about tick-borne pathogens and diseases? Choose all that apply

Check all that apply.

- [ ] From the media(Tv and radio)
- [ ] I read fro the news paper
- [ ] From the internet
- [ ] From a veterinary expert
- [ ] From a friend/family member

58. 1.2.13 Do you think that the tick causes diseases to the animal?

Mark only one oval.

- Yes, because the animals alway get sick whenever ticks are attached to it
- No, because the animal has never been sick even when ticks are found attched to it
- I don't know

59. 1.2.14 How often do you get veterinary services for your animals?

Mark only one oval.

- Never
- Occasionally (when I suspect something is wrong)
- Sometimes (only when they are sick)
- Often (regular routine checks)

60. 1.2.15 What type of diseases/infections/symptoms are the animals often diagnosed of? Choose all that apply

Check all that apply.

- [ ] Diarrhoea/watery droppings
- [ ] Running nose and teary eyes
- [ ] Dullness and inability to eat/Isolation from other animal and people
- [ ] Rushes and sores on the skin
- [ ] Pregnancy abortion
- [ ] None othe above
- [ ] Other: __________

61. 1.2.16 What type of tests are conducted to diagnose the disease on the animal?

Mark only one oval.

- ( ) Blood test
- ( ) Physical examination and prescribe drug for the animal
- ( ) No test is done. Veterinary officer just tells us the drug to buy for treatment
- ( ) No test is done. I just go to the pharmacy and buy a drug for my sick animals

62. 1.2.17 What kind of treatments are given to the animals when they are sick? Choose all that apply

Check all that apply.

- [ ] Antibiotics
- [ ] Antiviral vaccination
- [ ] Something to stop the diarrhoea
- [ ] Pain killers
- [ ] I only apply ointments to the wound and rushes
- [ ] I don't know about the drug

2.0 Tick, tick-borne diseases and humans

Public health wareness on tick-borne pathogens and diseases

63. 2.1 Have you been bitten by a tick before?

Mark only one oval.

- Yes. proceed to the next question
- No. skip the next 2 questions!
- Maybe

64. 2.2 What were the symptoms/signs of the tick bite? Choose all that apply

Check all that apply.

- [ ] Itching and irritations
- [ ] Swelling and rashes around the bitten area
- [ ] Fever and headache
- [ ] No symptoms at all

65. 2.3 What action did you take after taking the tick bite?

Mark only one oval.

- I went to the doctor/pharmacy to get tested for possible infections
- I did nothing about it because it did not hurt
- I hate tick but they do not cause any harm to human
- Other: ____________________

66. 2.4 Have you heard of tick-borne pathogens and disease in humans before?

Mark only one oval.

- Yes. proceed to the next question
- No, I have never heard of it. skip the next question
- I don't remember

67. 2.5 What type of tick-borne pathogens/diseases do you know of? Choose all that apply

Check all that apply.

- [ ] Bacteria (e.g. Borelia disease/lyme disease)
- [ ] Viral diseases (e.g tick-borne encephalitis-TBE, Cremean-congo virus-CCHV)
- [ ] Protozoa diseases
- [ ] Rickettsia diseases
- [ ] None of the above
- [ ] Other: ______________

68. 2.6 Do you think that humans can get diseases from tick bite?

Mark only one oval.

- Yes
- No
- Maybe

69. 2.7 Do you think you have ever gotten tick-borne diseases before without knowing?

Mark only one oval.

- Yes
- No
- Maybe

70. 2.8 Have you ever tested for tick-borne infections before?

Mark only one oval.

- Yes
- No

71. 2.9 Have you or any of your family members been diagnosed of tick-borne diseases before?

Mark only one oval.

- Yes
- No
- I have never heard of it

72. 2.10 If yes, what were the symptoms you experienced? Choose all that apply

Check all that apply.

- [ ] Diarrhoea/frequent watery stool
- [ ] Fever and headache
- [ ] Rashes and swelling on my body area
- [ ] Dizziness and loss of appettite
- [ ] No symptoms at all
- [ ] Other: ______________________

73. 2.11 Have you ever received treatments for suspected tick-borne diseases/infection)

Mark only one oval.

- Yes
- No

74. 2.12 If yes, what kind of treatment were you given/take?

Mark only one oval.

- Herbal medicine/formula
- Antibiotics prescribed by a doctor
- I did not take any medicine
- Self medication
- Pain killers

This content is neither created nor endorsed by Google.

Google Forms
