# Supplementary material for: Public knowledge and awareness of tick-borne pathogens and diseases: A cross-sectional study in Ghana
Source: Curr Res Parasitol Vector Borne Dis. 2024 Nov 4;6:100228. doi: 10.1016/j.crpvbd.2024.100228 (PMC11570981; doi:10.1016/j.crpvbd.2024.100228)
Supplement: Supplementary file S2 — Supplementary Figures and Tables. [file mmc2.pdf]

Supplementary file S2

Public knowledge and awareness of tick-borne pathogens and diseases: A cross-sectional study in Ghana

Theophilus Yaw Alale [a, b*], Jani J. Sormunen [a, b], Joseph Nzeh [c], Richard Osei Agjei [d], Eero J. Vesterinen [a], Tero Klemola [a]

[a] *Department of Biology, University of Turku, FI-20014 Turku, Finland*

[b] *Biodiversity Unit, University of Turku, FI-20014 Turku, Finland*

[c] *Department of Food Science and Technology, Kasetsart University, Bangkok, Thailand*

[d] *Department of Health Administration and Education, University of Education, P.O.Box 25, South Campus, Winneba, Ghana*

Contents

Supplementary Material A 1

1. Associations between level of education and awareness of TBDs/TBPs. 2
2. Associations between professional qualifications and awareness of TBDs/TBPs. 4
3. Associations between gender and awareness of TBDs/TBPs 5

Supplementary Material B ....

Table S1. Regional distribution of survey respondents from all 16 regions of Ghana. 7

Table S2. Gender distribution of respondents 7

Table S3. Age distribution of respondents 8

Table S4. Domestic animals kept by respondents. 9

Table S5. Respondents' sources of information about tick-borne pathogens/diseases 10

Table S6. Respondent access to veterinary services 11

Table S7. Potential tick-borne pathogens/diseases presented in the survey to respondents. 12

Associations between gender, level of education and professional qualification and TBDs awareness of both human and animal diseases.

1. Associations between level of education and awareness of TBDs/TBPs.

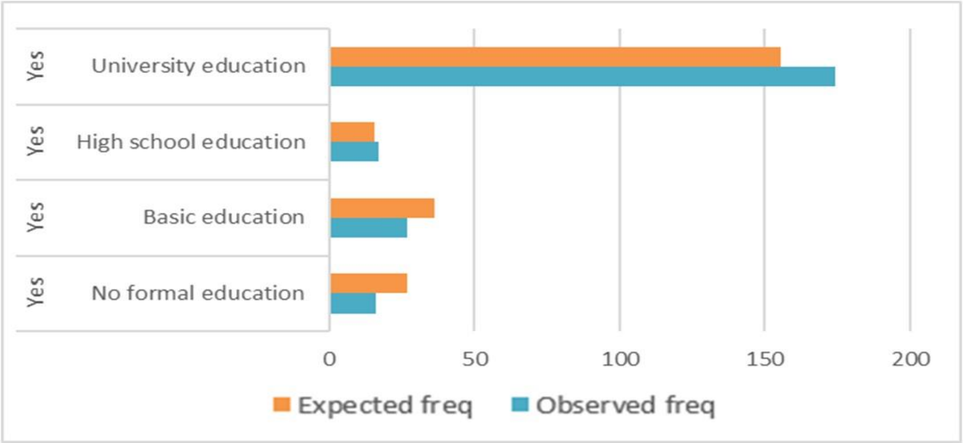

Supplementary Figure S1. Association between the education level of respondents and the question "Do you think that humans can get tick diseases from tick bite?" (answers "Yes" or "No"). Respondents with the university education were more and respondents with no formal or basic education less aware of possibility to get a tick disease from tick bite (χ^2 = 17.3, *df* = 3, *P* = 0.0006).

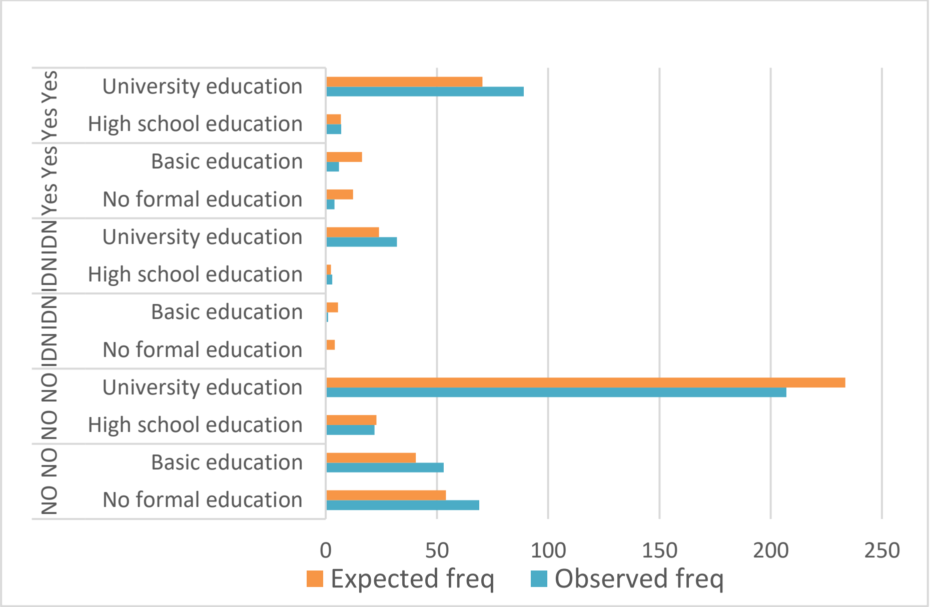

Supplementary Figure S2. Association between the education level of respondents and the question "Have you heard of tick-borne pathogens and diseases in humans before?" (answers "Yes", "I do not know" or "No"). Respondents with university education were more aware of TBPs/TBDs in humans than respondents with basic or no formal education.(χ^2 = 38.67, *df* = 6, *P* < 0.0001).

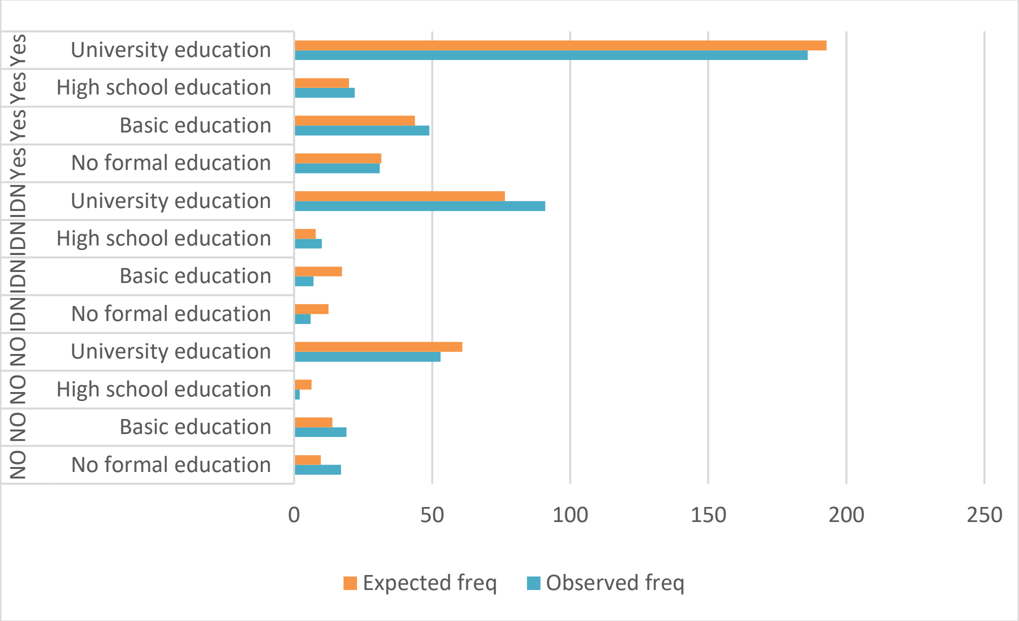

Supplementary Figure S3. Association between the education level of respondents and the question “Have you heard of tick-borne pathogens and diseases in animals?” (answers “Yes”, “I do not know” or “No”). Respondents with university level education were less aware of TBPs/TBDs in animals compared to those with basic education (χ^2 = 24.86, *df* = 6, *P* = 0.0004).

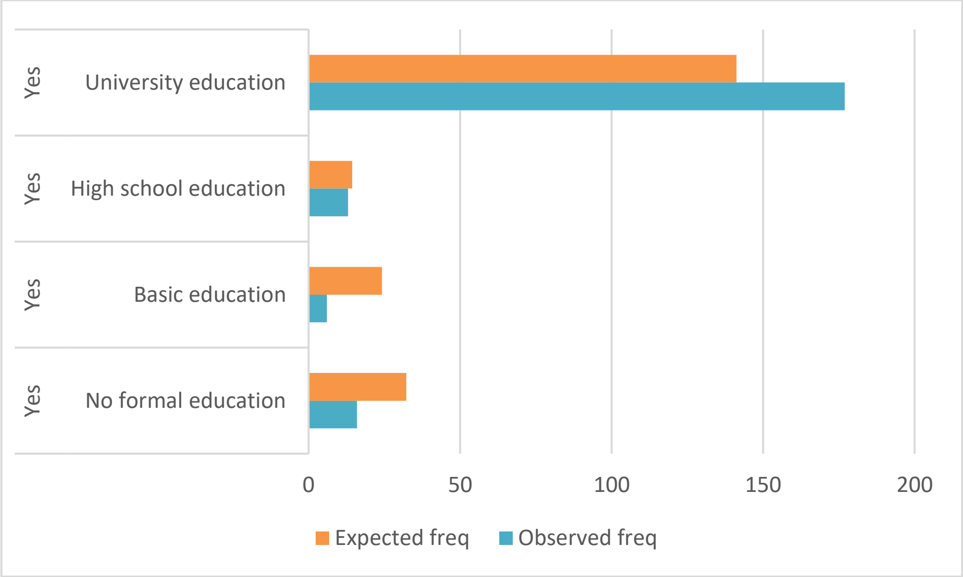

Supplementary Figure S4. Association between the education level of respondents and the question “Have you heard of tick-borne pathogens and diseases?” (answers “Yes” or “No”). Respondents with university-level education were more of TBDs/TBPs (χ^2 = 53.90, *df* = 3, *P* < 0.0001).

2. Associations between professional qualifications and awareness of TBDs/TBPs?

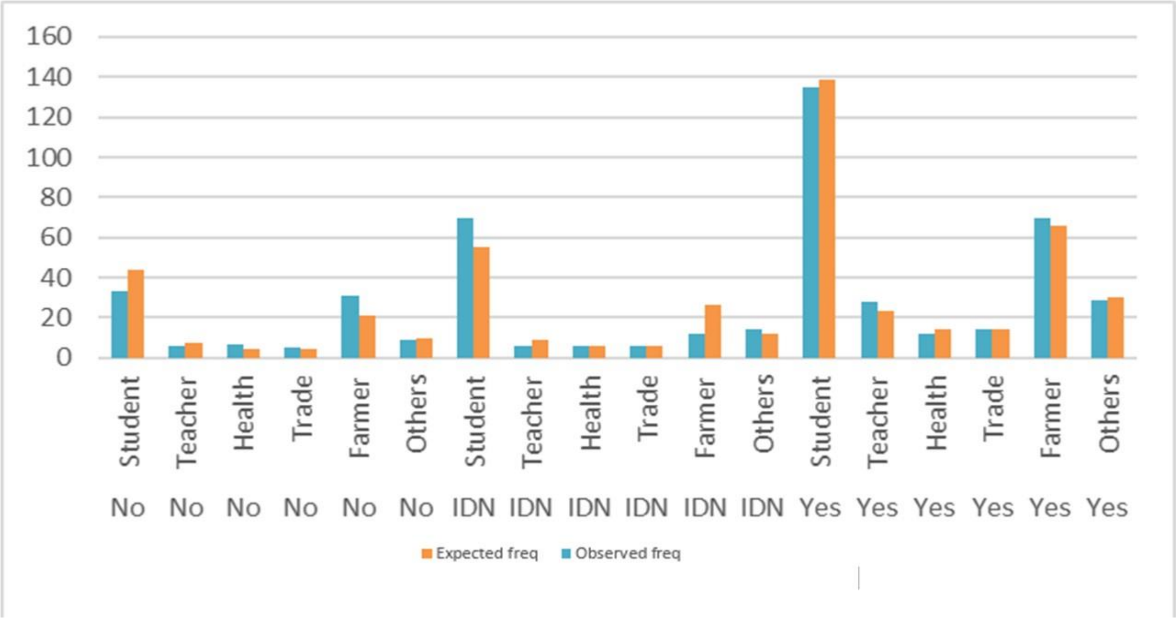

Supplementary Figure S5. Association between the professional qualification of respondents and the question "Do you think that ticks can cause diseases to animals?" (answers "Yes", "I do not know" or "No").

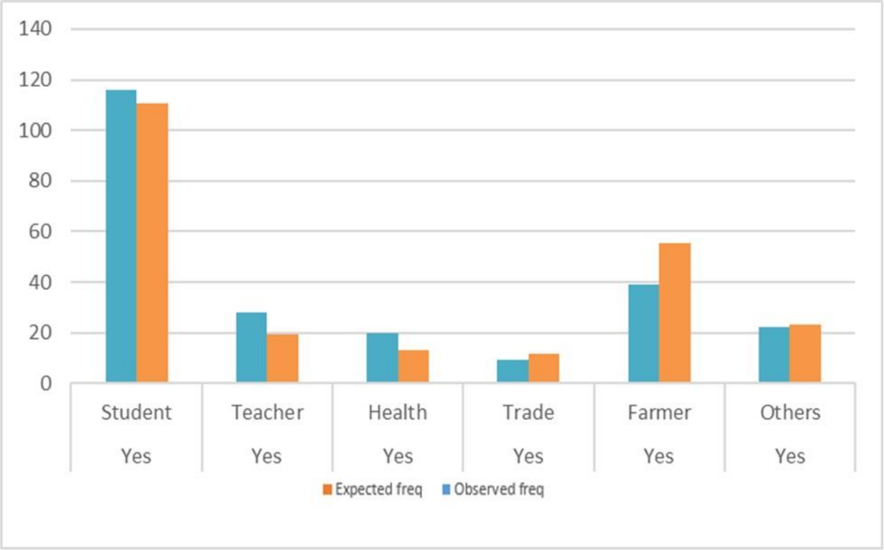

Supplementary Figure S6. Association between the professional qualification of respondents and the question "Do you think that humans can get disease from tick bite?" (answers "Yes" or "No"). Farmers were the least aware of human#s getting diseases from ticks (χ^2 = 24.81, *df* =5, *P* = 0.0002).

3. Associations between gender and awareness of TBDs/TBPs?

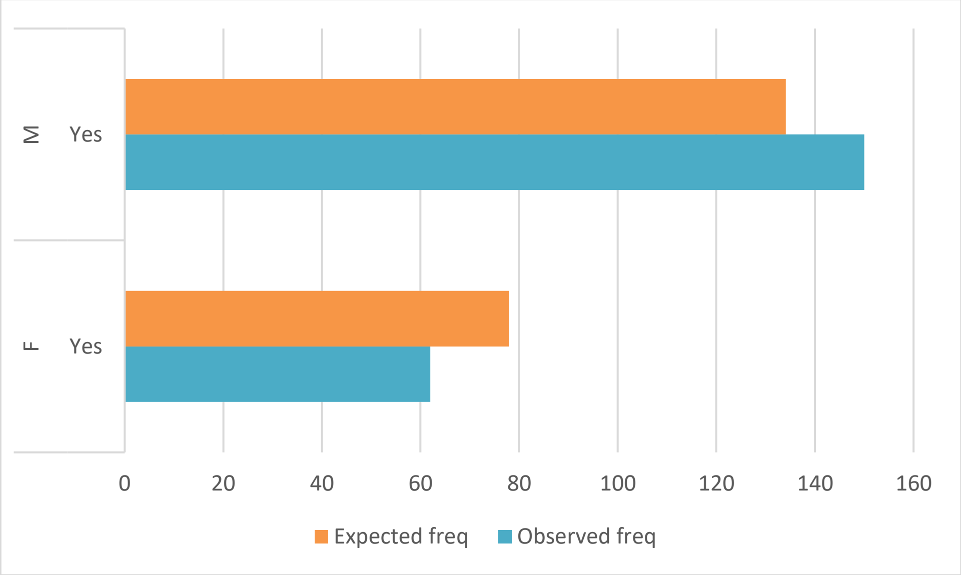

Supplementary Figure S7. Association between the gender of respondents and the question "Have you heard of tick-borne pathogens and diseases?" (answers "Yes" or "No"). Male respondents were more aware of TBPs/TBDs than females (χ^2 = 8.94, *df* =1, *P* = 0.003).

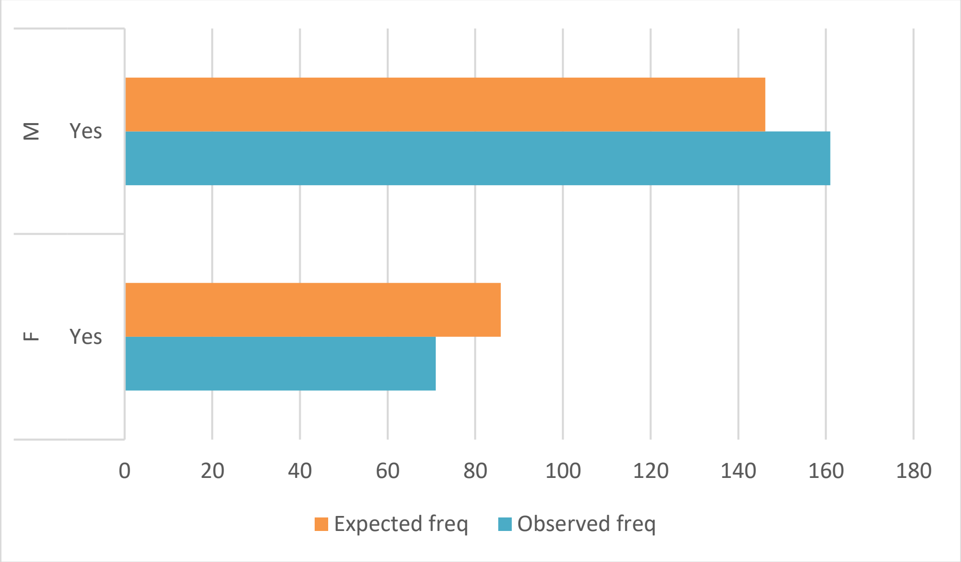

Supplementary Figure S8. Association between the gender of respondents and the question "Do you think humans can get diseases from tick bite?" (answers "Yes" or "No").

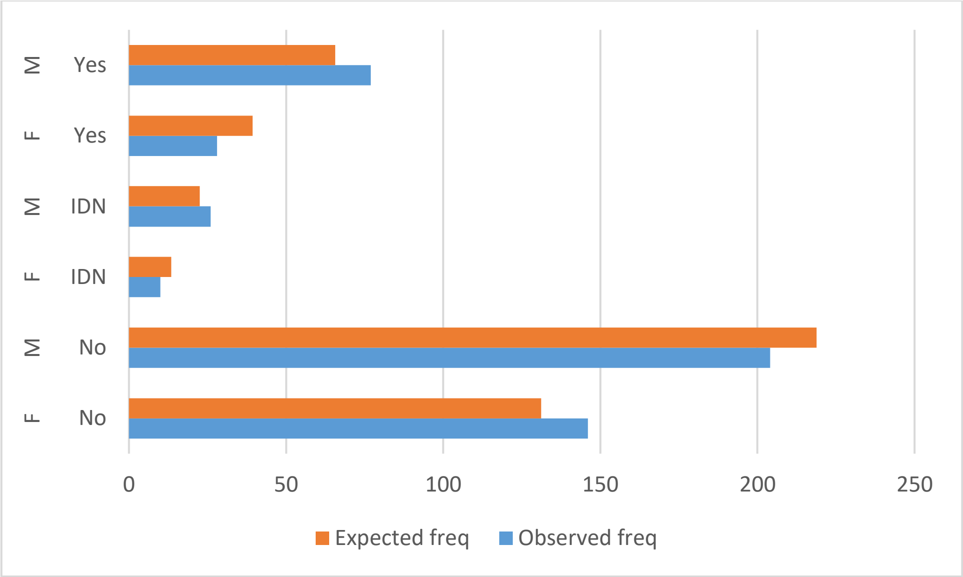

Supplementary Figure S9. Association between the gender of respondents and the question "Have you heard of TBDs/TBPs in human before?" (answers "Yes", "I do not know" or "No"). More male respondents had heard of TBDs/TBPs in humans before (χ^2 = 9.36, *df* =2, *P* = 0.009).

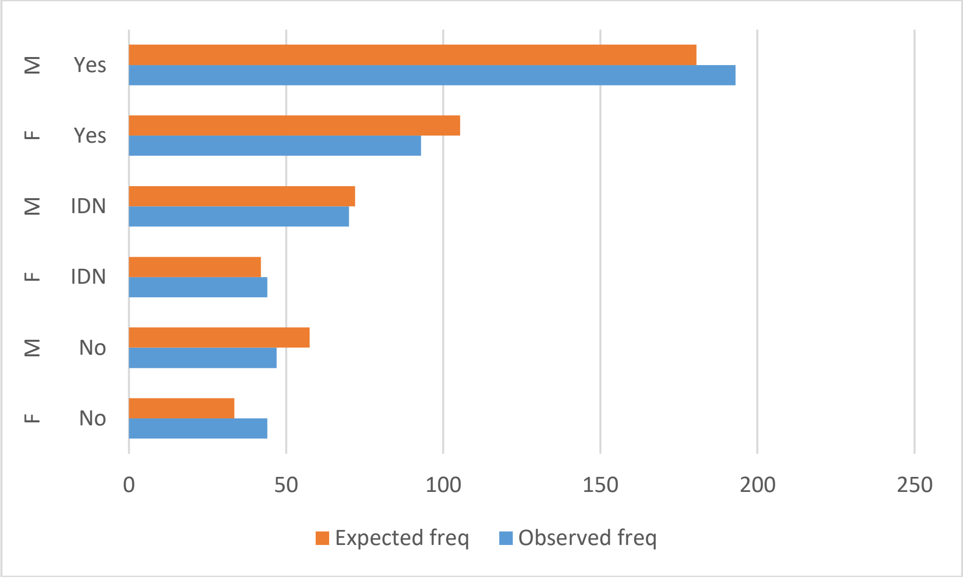

Supplementary Figure S10. Association between the gender of respondents and the question "Have you heard of TBDs/TBPs in animals?" (answers "Yes", "I do not know" or "No") Male respondents were more and female respondents less aware that TBPs can cause diseases in animals (χ^2 = 7.62, DF =2, p = 0.02).

Supplementary Table S1. Regional distribution of survey respondents from all 16 regions of Ghana.

Regions in Ghana	Frequency	Percent	Cumulative Frequency	Cumulative Percent
Ahafo region	6	1.13	6	1.13
Ashanti region	133	24.95	139	26.08
Bono East region	7	1.31	146	27.39
Bono region	8	1.50	154	28.89
Central rgion	24	4.50	178	33.40
Eastern region	12	2.25	190	35.65
Greater Accra region	66	12.38	256	48.03
North East region	4	0.75	260	48.78
Northern region	98	18.39	358	67.17
Oti region	1	0.19	359	67.35
Savannah region	6	1.13	365	68.48
Upper East rgion	8	1.50	373	69.98
Upper West rgion	6	1.13	379	71.11
Volta region	145	27.20	524	98.31
Western North region	3	0.56	527	98.87
Western region	6	1.13	533	100.00
Frequency Missing = 4				

Supplementary Table S2. Gender distribution of respondents

Sex	Frequency	Percent	Cumulative Frequency	Cumulative Percent
Female	200	37.52	200	37.52
Male	333	62.48	533	100.00
Frequency Missing = 4				

Supplementary Table S3. Age distribution of respondents

Your Age	Frequency	Percent	Cumulative Frequency	Cumulative Percent
**18-24**	222	41.65	222	41.65
**25-34**	184	34.52	406	76.17
**35-44**	65	12.20	471	88.37
**45-54**	45	8.44	516	96.81
**55+**	17	3.19	533	100.00
**Frequency Missing = 4**				

Supplementary Table S4. Domestic animals kept by respondents.

Which of the following animals do you keep at home? Choose all that apply				
**Animals**	**Frequency**	**Percent**	**Cumulative**	**Cumulative**
Cat(s)	49	9.14	49	9.14
Cat(s);Chicken	40	7.46	89	16.60
Cat(s);Chicken;Cattle	2	0.37	91	16.98
Cat(s);Chicken;Goat/Sheep	11	2.05	102	19.03
Cat(s);Chicken;Goat/Sheep;Cattle	4	0.75	106	19.78
Cat(s);Goat/Sheep	2	0.37	108	20.15
Cattle	3	0.56	111	20.71
Chicken	81	15.11	192	35.82
Chicken;Cattle	6	1.12	198	36.94
Chicken;Goat/Sheep	12	2.24	210	39.18
Chicken;Goat/Sheep;Cattle	1	0.19	211	39.37
Chicken;None	1	0.19	212	39.55
Dog(s)	76	14.18	288	53.73
Dog(s);Cat(s)	42	7.84	330	61.57
Dog(s);Cat(s);Cattle	1	0.19	331	61.75
Dog(s);Cat(s);Chicken	25	4.66	356	66.42
Dog(s);Cat(s);Chicken;Cattle	2	0.37	358	66.79
Dog(s);Cat(s);Chicken;Goat/Sheep	28	5.22	386	72.01
Dog(s);Cat(s);Chicken;Goat/Sheep;Cattle	22	4.10	408	76.12
Dog(s);Cat(s);Chicken;Goat/Sheep;Cattle;Donkey, pigs	1	0.19	409	76.31
Dog(s);Cat(s);Chicken;Goat/Sheep;None	1	0.19	410	76.49
Dog(s);Cat(s);Goat/Sheep	2	0.37	412	76.87
Dog(s);Cattle	2	0.37	414	77.24
Dog(s);Chicken	21	3.92	435	81.16
Dog(s);Chicken;Cattle	2	0.37	437	81.53
Dog(s);Chicken;Goat/Sheep	11	2.05	448	83.58
Dog(s);Chicken;Goat/Sheep;Cattle	6	1.12	454	84.70
Dog(s);Goat/Sheep	3	0.56	457	85.26
Dog(s);Goat/Sheep;Cattle	1	0.19	458	85.45
Goat/Sheep	11	2.05	469	87.50
Guinea f	1	0.19	470	87.69
None	62	11.57	532	99.25
None;Used to have a dog	1	0.19	533	99.44
Peaks	1	0.19	534	99.63
Rabbit	1	0.19	535	99.81
Snails	1	0.19	536	100.00
Frequency Missing = 1				

Supplementary Table S5. Respondents' sources of information about tick-borne pathogens/diseases

From where did you hear about tick-borne pathogens and diseases? Choose all that apply				
**Information sources**	**Frequency**	**Percent**	**Cumulative Frequency**	**Cumulative Percent**
**From a friend/family member**	49	16.44	49	16.44
**From a veterinary expert**	66	22.15	115	38.59
**From a veterinary expert;From a friend/family member**	8	2.68	123	41.28
**From the internet**	60	20.13	183	61.41
**From the internet;From a friend/family member**	5	1.68	188	63.09
**From the internet;From a veterinary expert**	9	3.02	197	66.11
**From the internet;From a veterinary expert;From a friend/family member**	3	1.01	200	67.11
**From the media(Tv and radio)**	50	16.78	250	83.89
**From the media(Tv and radio);From a friend/family member**	5	1.68	255	85.57
**From the media(Tv and radio);From a veterinary expert**	5	1.68	260	87.25
**From the media(Tv and radio);From a veterinary expert;From a friend/family member**	3	1.01	263	88.26
**From the media(Tv and radio);From the internet**	9	3.02	272	91.28
**From the media(Tv and radio);From the internet;From a friend/family member**	6	2.01	278	93.29
**From the media(Tv and radio);From the internet;From a veterinary expert**	2	0.67	280	93.96
**From the media(Tv and radio);From the internet;From a veterinary expert;From a friend/family member**	2	0.67	282	94.63
**From the media(Tv and radio);I read fro the news paper;From the internet;From a friend/family member**	2	0.67	284	95.30
**From the media(Tv and radio);I read fro the news paper;From the internet;From a veterinary expert;From a friend/family member**	7	2.35	291	97.65
**I read fro the news paper**	4	1.34	295	98.99
**I read fro the news paper;From a veterinary expert;From a friend/family member**	2	0.67	297	99.66
**I read fro the news paper;From the internet;From a veterinary expert**	1	0.34	298	100.00
**Frequency Missing = 239**				

Supplementary Table S6. Respondent access to veterinary services.

How often do you get veterinary services for your animals?				
**Category**	**Frequency**	**Percent**	**Cumulative Frequency**	**Cumulative Percent**
**Never**	224	46.38	224	46.38
**Occasionally (when I suspect something is wrong)**	125	25.88	349	72.26
**Often (regular routine checks)**	42	8.70	391	80.95
**Sometimes (only when they are sick)**	92	19.05	483	100.00
**Frequency Missing = 54**				

Supplementary Table S7. Potential tick-borne pathogens/diseases presented in the survey to respondents.

What type of tick-borne pathogens/diseases do you know of? Choose all that apply				
**Potential tick-borne pathogen/diseases**	**Frequency**	**Percent**	**Cumulative Frequency**	**Cumulative Percent**
**Bacteria (e.g. Borelia disease/lyme disease)**	47	21.27	47	21.27
**Bacteria (e.g. Borelia disease/lyme disease);Protozoa diseases**	5	2.26	52	23.53
**Bacteria (e.g. Borelia disease/lyme disease);Protozoa diseases;Rickettsia diseases**	1	0.45	53	23.98
**Bacteria (e.g. Borelia disease/lyme disease);Viral diseases (e.g tick-borne encephalitis-TBE, Cremean-congo virus-CCHV)**	6	2.71	59	26.70
**Bacteria (e.g. Borelia disease/lyme disease);Viral diseases (e.g tick-borne encephalitis-TBE, Cremean-congo virus-CCHV);Protozoa diseases**	13	5.88	72	32.58
**Bacteria (e.g. Borelia disease/lyme disease);Viral diseases (e.g tick-borne encephalitis-TBE, Cremean-congo virus-CCHV);Protozoa diseases;Rickettsia diseases**	8	3.62	80	36.20
**Bacteria (e.g. Borelia disease/lyme disease);Viral diseases (e.g tick-borne encephalitis-TBE, Cremean-congo virus-CCHV);Protozoa diseases;Rickettsia diseases;None of the**	1	0.45	81	36.65
**Bacteria (e.g. Borelia disease/lyme disease);Viral diseases (e.g tick-borne encephalitis-TBE, Cremean-congo virus-CCHV);Rickettsia diseases**	3	1.36	84	38.01
**Kill the tick**	1	0.45	85	38.46
**Lyme disease**	1	0.45	86	38.91
**None of the above**	82	37.10	168	76.02
**Protozoa diseases**	17	7.69	185	83.71
**Protozoa diseases;Rickettsia diseases**	2	0.90	187	84.62
**Rickettsia diseases**	5	2.26	192	86.88
**Viral diseases (e.g tick-borne encephalitis-TBE, Cremean-**	25	11.31	217	98.19
**Viral diseases (e.g tick-borne encephalitis-TBE, Cremean-**	4	1.81	221	100.00
**Frequency Missing = 316**				
